# Signal transducer and activator of transcription 3 (STAT3) inhibitor, S3I-201, acts as a potent and non-selective alkylating agent

**DOI:** 10.18632/oncotarget.7838

**Published:** 2016-03-02

**Authors:** Daniel P. Ball, Andrew M. Lewis, Declan Williams, Diana Resetca, Derek J. Wilson, Patrick T. Gunning

**Affiliations:** ^1^ Department of Chemical and Physical Sciences, University of Toronto Mississauga, Mississauga, Ontario, L5L 1C6, Canada; ^2^ Department of Chemistry, York University, Toronto, Ontario, M3J 1P3, Canada; ^3^ Department of Chemistry, Center for Research in Mass Spectrometry, York University, Toronto, Ontario, M3J 1P3, Canada; ^4^ Department of Medical Biophysics, University of Toronto, Toronto, Ontario, M5G 1L, Canada

**Keywords:** STAT3, S3I-201, NSC 74859, covalent modification, oncology

## Abstract

The Signal Transducer and Activator of Transcription 3 (STAT3) oncogene is a master regulator of many human cancers, and a well-recognized target for therapeutic intervention. A well known STAT3 inhibitor, S3I-201 (NSC 74859), is hypothesized to block STAT3 function in cancer cells by binding the STAT3 SH2 domain and disrupt STAT3 protein complexation events. In this study, liquid chromatography tandem mass spectrometry analysis revealed that STAT3, in the presence of S3I-201, showed a minimum of five specific sites of modification, cysteine's 108, 259, 367, 542, and 687. Moreover, a prepared fluorescently labeled chemical probe of S3I-201 (DB-6-055) revealed that S3I-201 non-specifically and globally alkylated intracellular proteins at concentrations consistent with S3I-201's reported IC_50_. These data are consistent with the hypothesis that S3I-201 is a sub-optimal probe for interrogating STAT3-related cell biology.

## INTRODUCTION

Signal Transducer and Activator of Transcription 3 (STAT3) protein, a cytosolic protein, has been identified as a key regulator of human cancers, contributing to uncontrolled differentiation, proliferation, survival, and tumorigenesis. For example, STAT3 is found to be active in many cancers including breast, gastric, brain, and lung [1–4]. As a result, much effort has been expended to identify inhibitors of the active motif, a STAT3:STAT3 homo-dimer, which is able to bind DNA and promote the transcription of STAT3 target genes. The STAT3:STAT3 dimer complex is facilitated by reciprocal binding interactions between the SH2 domain of one STAT3 and the phosphorylated tyrosine residue (pY705) of another STAT3 protein. Mimicking the pY-containing binding epitope has afforded inhibitors capable of disrupting the STAT3 protein–protein interaction and attenuating its aberrant activity [5]. These focused molecular efforts have included peptidomimetic and small molecule mimicry of the pY sequence, as well as coordination complexes mimicking the SH2 domain [6]. Of note, Stattic [7], S3I-201 (NSC74859) [8], niclosamide [9], and a number of other small molecules were identified as being potential lead points for drug development [10–12]. Our own efforts, building on the pY-mimicking salicylic acid motif of S3I-201, have yielded more potent small molecule STAT3 inhibitors, SF-1-066, BP-1-102, SH-4-054, and most recently, BP-5-087 [13–18]. Several of these compounds, including S3I-201, BP-1-102 and SH-4-054 have advanced to preclinical trials in a number of human diseases. Given that these inhibitors possessed electrophilic sites, susceptible to nucleophilic attack, it was hypothesized that the efficacy demonstrated *in vivo* might be a result of covalent modification of target proteins and/or other targets. We first considered the electrophilic *O*-tosyl group of S3I-201 to be problematic in 2007. This issue was addressed by Fletcher *et al*. [14], wherein the oxygen atom of the *O*-tosyl group was replaced with a stable nitrogen sulfonamide derivative. The O to NH/N-Me replacement dramatically reduced the inhibition of activated STAT3 dimerization from 86 μM to > 300 μM (STAT3 EMSA assay), suggesting that the *O*-tosyl group was required for activity. While recognized as a reactive electrophile, the *O*-tosyl group has been used for selective modification of protein targets in a cellular environment. For example, Hamachi has pioneered the field of ligand-directed *O*-tosyl (LDT) chemistry, attaching fluorescent probes onto specific nucleophilic amino acid residues of proteins of interest [19,20]. These experiments have demonstrated that *O*-tosyl-containing molecules can be utilized as protein-selective chemical probes. However, it is important to fully understand the targets of such molecules to ensure proper interpretation of experimental results in complex cellular systems.

Herein, we report/characterize the reactivity profile of S3I-201, BP-1-102, and SH-4-54 with the biological nucleophile GSH. Digestive LC-MS/MS fragmentation spectroscopy, and a chemical biology approach are employed to determine whether S3I-201 acts as a targeted covalent inhibitor of activated STAT3. Using a fluorescent probe of S3I-201, the effect on STAT3 protein, as well as the global effect on MDA-MB-231 cancer cells was determined. We present data to suggest that S3I-201 is a strong covalent modifier of many cellular proteins and is unsuitable for use as a STAT3-selective chemical probe.

## RESULTS

### S3I-201's reactivity toward glutathione

**Figure 1:**
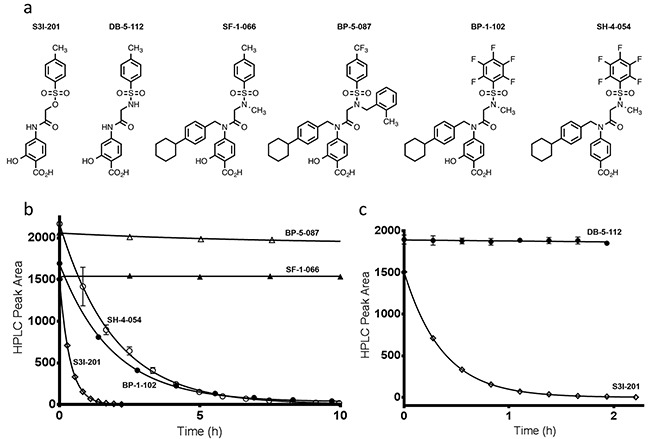
**a.** Chemical structures of S3I-201, DB-5-112, SF-1-066, BP-5-087, BP-1-102, and SH-4-054. **b.** Reaction time course for the decay in HPLC peak area for BP-5-087, SF-1-066, SH-4-054, BP-1-102, and S3I-201 in the presence of 10 mM GSH, 10 mM TCEP-HCl and 50 mM HEPES pH 7.4 (fitted parameters available in Supplementary Table S2). **c.** Shows the reactivity differences between the *O*-tosyl group of S3I-201 and the sulfonamide analogue DB-5-112 under identical reaction conditions, over the same reaction time course (fitted parameters available in Supplementary Table S3).

**Figure 2:**
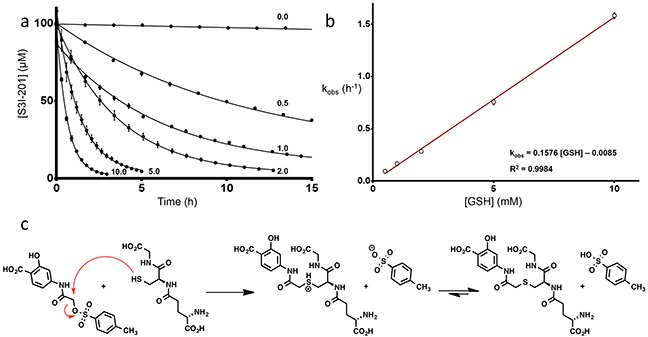
**a.** Reactivity time courses for the decay of 100 μM S3I-201 in the presence of increasing concentrations of GSH (0.5, 1.0, 2.0, 5.0 10.0 mM) in a 100 mM HEPES buffer, pH 7.4 (fitted parameters available in Supplementary Table S4). **b.** Plot of the observed pseudo-first order rate constants (k_obs_) for the reaction of S3I-201 at increasing concentrations of GSH (0.5, 1.0, 2.0, 5.0, 10.0 mM) as a function of its concentration (fitted parameters available in Supplementary Table S5). **c.** Proposed operative mechanism of reaction between GSH and S3I-201 consistent with first order dependence in GSH concentration.

An HPLC-based assay was developed to determine compound stability to the nucleophile, glutathione (GSH), which is abundant in hepatocyte cells (7.5 mM) [21]. Initial studies were conducted with 100 μM of S3I-201, BP-1-102, SH-4-054, BP-5-087, and SF-1-066 with a 100-fold excess of GSH (structures shown in Figure 1a). 5-fold faster GSH reaction times with S3I-201 were observed as compared to BP-1-102, and SH-4-054 within the 10 h timescale tested (Figure 1b). SH-4-054 and BP-1-102, containing the nucleophilic aromatic substitution-susceptible pentafluorobenzene sulfonamide substituent [22–25], exhibited reaction half lives (t_1/2_) of 1.35 and 1.30 h, respectively. BP-5-087 and SF-1-066, not containing an identifiable electrophile showed negligible reactivity with GSH. S3I-201, containing an *O*-tosyl functional group was rapidly degraded under the same assay conditions, with a t_1/2_ of 0.25 h, a ∼ 5-fold greater rate as compared to pentafluorobenzene sulfonamide-containing compounds. As a control, the same experiment was conducted with the sulfonamide analog of S3I-201, DB-5-112 (Figure 1a and 1c, synthesis in Supplementary information). Under the same reaction conditions, DB-5-112 showed no reactivity toward GSH (10 mM). Given these results, the chemical reactivity of S3I-201 was investigated further. To determine the reaction order with respect to GSH, a concentration-dependent pseudo-first order reactivity study was conducted (Figure 2a). TCEP-HCl was removed from the reaction buffer for these studies, as the oxidation of GSH to GSSG did not occur competitively during the time scale tested and buffered solutions were freshly prepared prior to each experiment. The observed pseudo-first order rate constants obtained (0.5 mM ≤ [GSH] ≤ 10.0 mM) were plotted as a function of the GSH concentration. The resultant linear slope (Figure 2b) provided the bimolecular rate constant of the reaction between S3I-201 and GSH (k_2_ = 0.158 ± 0.004 h^−1^mM^−1^) and produced strong evidence of a first order reaction dependence with respect to the nucleophile, GSH. This result is consistent with a bimolecular, nucleophilic substitution reaction mechanism (S_N_2), shown in Figure 2c.

### Incubation of STAT3 with S3I-201, tryptic digestion and LC-MS/MS analysis

**Figure 3:**
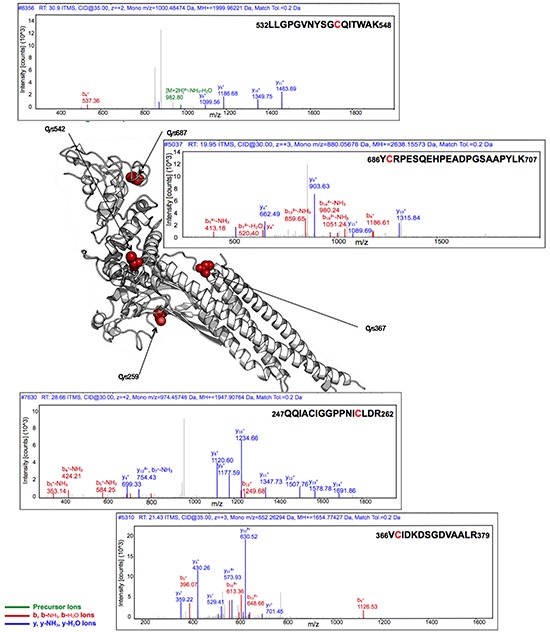
Covalent modification of full-length STAT3 by S3I-201 as analyzed by LC-MS/MS Top upper trace provides the fragment ion analysis of the peptide sequence 532LLGPGVNYSGCQITWAK548 for the determination of the alkylation of Cys542. Top lower trace provides the fragment ion analysis of the peptide sequence 686YCRPESQEHPEADPGSAAPYLK707 for the determination of the alkylation of Cys687. Bottom upper trace provides the fragment ion analysis of the peptide sequence 247QQIACIGGPPNICLDR262 for the determination of the alkylation of Cys259. Bottom lower trace provides the fragment ion analysis of the peptide sequence 366VCIDKDSGDVAALR379 for the determination of the alkylation of Cys367. The central image was generated on PyMol image software using PDB: 4E68 where modified Cys residues are highlighted in red. Cys108, also shown alkylated by S3I-201 is unresolved in PDB code 4E68 and is thus excluded from the figure above. The LC-MS/MS analysis for Cys108 is found in the Supplementary Figure S2 and extensive list of identified MS/MS fragment ions found in Supplementary Tables S6, S7, S8, S9, and S10.

S3I-201 was next assessed for its reactivity with STAT3. Recombinant, full-length STAT3 protein was incubated with S3I-201, and after reduction and alkylation of free sulfhydryl groups with iodoacetamide, was digested with trypsin and analyzed by LC-MS/MS for the presence of S3I-201-modified species. Subjected to incubation with an 80-fold molar excess of S3I-201, the full-length STAT3 protein was found to be covalently modified (Figure 3). MS/MS sequencing of the tryptic peptides demonstrated that S3I-201 covalently modified Cys108, Cys259, Cys367, Cys542, and Cys687 of STAT3 in a manner consistent with thiol-mediated *O*-tosyl substitution. For example, the peptide sequence containing Cys542 of STAT3 (532LLGPGVNYSGCQITWAK548) was identified following digestion along with a multitude of notable mass fragments characterized as either b_i_^+^ or y_i_^+^ ion fragments. y_i_^+^ ions are those whose fragments extend from the C-terminus of a given peptide fragment and b_i_^+^ ions are those analogous ions from the N-terminus. Therefore, a y_i_^+^ ion fragment containing 9 residues from the C-terminus (y_9_^+^) would give 539SGCQITWAK548 (positive ion mass of 993.483 Da) and contain a potentially reactive cysteine residue. Under the proposed mechanism of covalent modification, S3I-201 would add a mass fragment of 194.04 Da. This would suggest that a covalent adduct for a y_9_^+^ ion would be equal to approximately 1186.523 Da (after subtracting the proton from the sulfhydryl of cysteine). Figure 3 (top panel) showed a mass fragment of 1186.68 *m/z*, consistent with the attachment of an S3I-201 conjugate to the sequence. Evidence of y_8_^+^, y_10_^+^, y_11_^+^ ions all support the hypothesis of the formation of covalent adducts. Conversely, a relevant example of a b_i_^+^ ion fragment would originate from the N-terminus of the sequence. Thus, if you consider 686YCRPESQEHPEADPGSAAPYLK707 a b_8_^+^ fragment ion corresponds to the sequence 686YCRPESQE693 (positive ion mass of 993.4101 Da). An S3I-201 adduct of this species would give an ion mass of 1186.580 Da and was observed 1186.61 *m/z* along with other similarly modified mass fragments. In its totality, Figure 3 provides significant evidence of numerous alkylation events by S3I-201 onto multiple cysteine residues of STAT3 (MS/MS fragment spectrum can be found in Supplementary Figure S2 and a complete list of observed fragment ions can be found in Supplementary Tables S6, S7, S8, S9, and S10). Further modification of the remaining exposed cysteine residues cannot be excluded. However, evidence of five positions of modification was sufficient to raise the question of inhibitor promiscuity.

### Design, synthesis, and biological evaluation of a fluorescently tagged analog of S3I-201

**Scheme 1:**
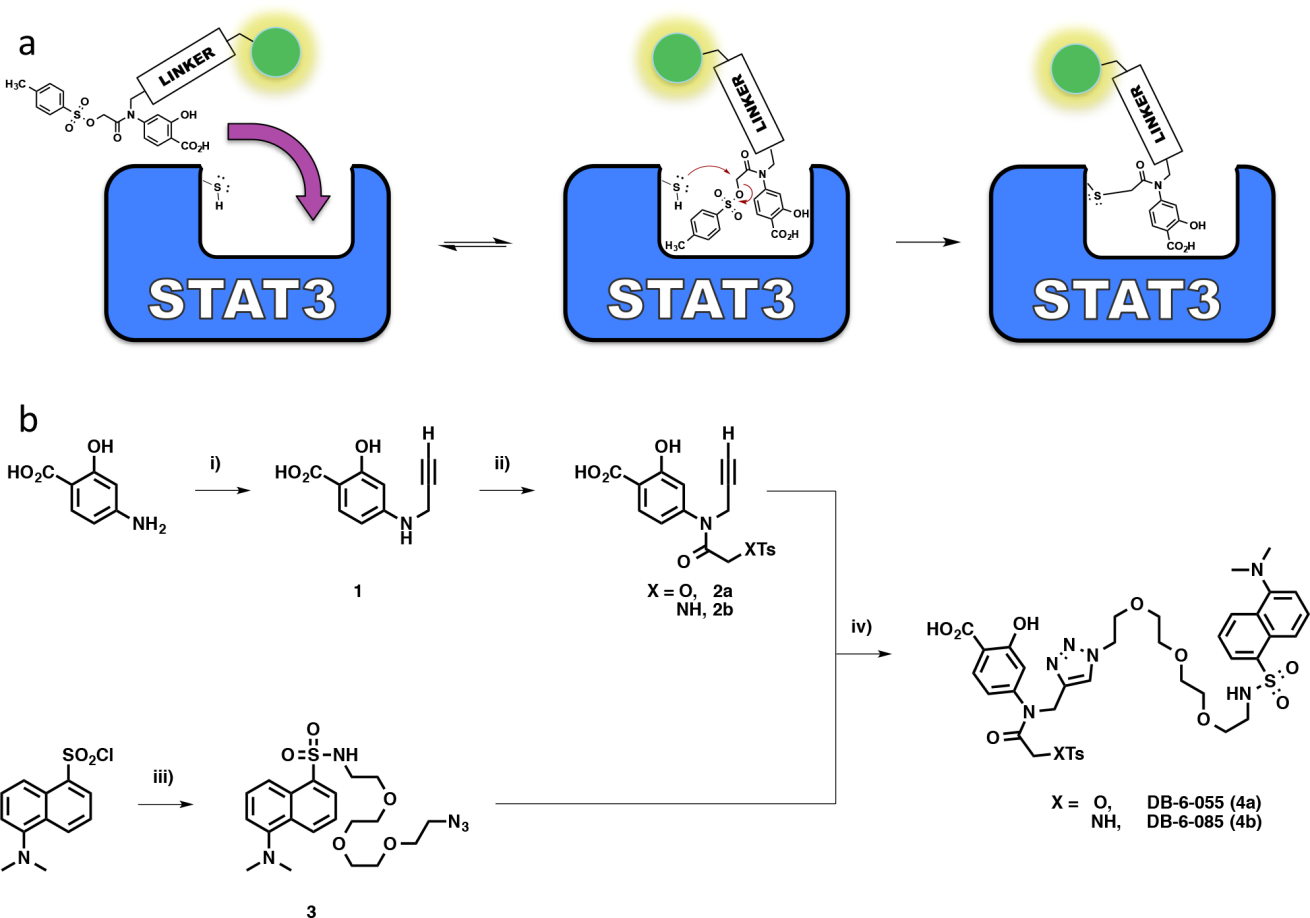
**a.** Schematic diagram for a fluorescently tagged S3I-201 molecule and subsequent hypothesized mode of action supported by kinetic data. **b.** Chemical synthesis of triazole-linked S3I-201 fluorescent reporter molecule 4a (Where X = O) or the sulfonamide analog, 4b (Where X = NH): i) Cs_2_CO_3_, 80 wt% propargyl bromide, DMF, 0°C – rt; ii) 1) Na_2_CO_3_, THF; 2) 2-Chloro-2-oxoethyl 4-methylbenzenesulfonate or tosylglycine, THF; 3) DIPEA/H_2_O; iii) 11-azido-3,6,9-trioxaundecane-1-amine, CH_2_Cl_2_, DIPEA, 0°C – rt; iv) CuSO_4_, sodium ascorbate, H_2_O/THF, rt.

To investigate further, a DANSYL fluorescent tag was appended to the structure of S3I-201, producing the chemical probe DB-6-055 (4a), to determine the extent of modification via a gel-based fluorescent read-out (chemical approach shown schematically in Scheme 1a). DANSYL was chosen for its known and predictable fluorescence behavior, solvatochromic properties, and compatibility with biological systems [26]. Covalent modification would render the protein visible by simple fluorescence imaging following denaturing, SDS-PAGE experiments. Since *in silico* molecular modeling revealed that S3I-201's amide nitrogen N-H group projected into the aqueous environment [8], we elected to attach the fluorophore from this position via propargylation to the tertiary amide followed by copper (II) sulfate (CuSO_4_) mediated azide-alkyne click chemistry with the DANSYL-tagged fluorescent azide.

**Figure 4:**
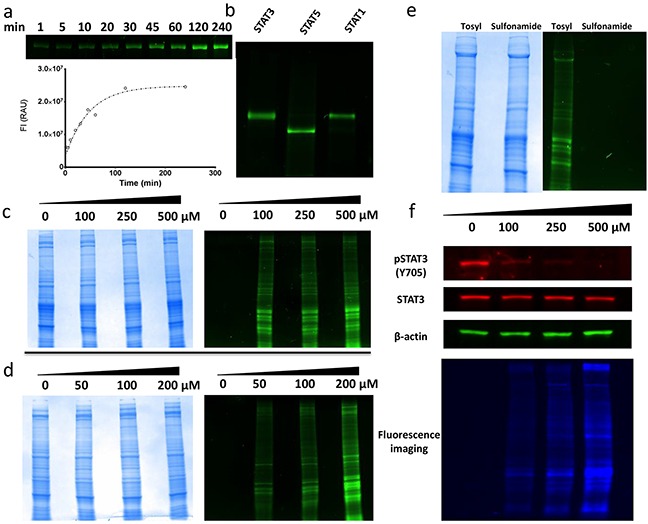
Molecular biology experiments for fluorescent probe molecules 4a and 4b **a.** Curve of 4a labeling purified STAT3 protein over a kinetic time course. SDS-PAGE image shows time-dependent increases in fluorescent intensities of STAT3 protein. The curve generated was obtained from the quantification of the relative fluorescent intensity of the respective bands within the gel. **b.** Purified STAT3, STAT5 and STAT1 proteins incubated with 100 μM 4a for 16 h followed by SDS-PAGE. **c.** Total protein images of SDS-PAGE for MDA-MB-231 cell lysates incubated with increasing doses of 4a. Total protein content observed by Coomassie Brilliant Blue staining (LEFT). Same gel viewed under fluorescent excitation conditions (RIGHT). **d.** Total protein images of SDS-PAGE for MDA-MB-231 cells dosed with increasing concentration of 4a prior to lysing. Total protein content observed by Coomassie Brilliant Blue staining (LEFT). Same gel viewed under fluorescent excitation conditions (RIGHT). **e.** SDS-PAGE of MDA-MB-231 cell lysates treated with either 4a (*O*-tosyl) or 4b (sulfonamide). Total protein content of samples visualized with Coomassie Brilliant Blue staining (LEFT). Same gel viewed under fluorescent excitation (RIGHT). **f.** Immunobloting for total STAT3 and activated pSTAT3 levels in MDA-MB-231 cells treated with increasing concentrations of 4a. Also shown is the same gel under fluorescent excitation conditions highlighting extent of total protein labeling.

First, to replicate the covalent modification observed in the MS/MS experiments with S3I-201, we incubated purified STAT3 protein with 100 μM of 4a (the approximate IC_50_ for the disruption of STAT3:DNA binding by S3I-201) over a time course (from 0-4 h). Reactions were quenched with a denaturing β-mercaptoethanol solution and analyzed by SDS-PAGE. As can be seen in Figure 4a, 4a induced a time dependent increase in fluorescent signal of the STAT3 protein indicative of covalent modification by 4a. Moreover, within the time course tested, fluorescent levels appeared to plateau. This suggested a 4a-mediated saturation of the STAT3 nucleophilic residues.

To probe for selectivity, 4a (100 μM) was incubated with STAT3, STAT5, and STAT1 purified proteins. The resulting gels provided clear evidence of non-specific fluorescent labeling of all three STAT isoforms (Figure 4b). Thus, it can be inferred from the 4a data that S3I-201 cannot discriminate between closely related STAT proteins at approximate IC_50_ concentrations of S3I-201. To further evaluate the selectivity of 4a in a more diverse biochemical environment, 4a was incubated with MDA-MB-231 breast cancer epithelial cell lysates at various concentrations (0, 100, 250, and 500 μM), followed by SDS-PAGE. As can be seen in Figure 4c, relative to the Coomassie Brilliant Blue total protein staining, treatment with 4a resulted in pronounced, non-specific, global labeling of the protein content within the sample. Furthermore, with increasing doses of 4a, higher fluorescent intensities of the banding patterns was observed.

Next, for assessing cell-penetrating properties, MDA-MB-231 whole cells were treated with increasing concentrations of 4a (0, 50, 100, and 200 μM). As Figure 4d shows, analogous labeling of the lysates occurred, with increasing levels of fluorescent intensities following the dose of reporter. Taken together, these results supported the hypothesis that S3I-201 covalently modifies not only the STAT family of proteins but alkylates a large number of proteins within the cell in a seemingly unselective manner.

As an unreactive fluorescent control, DB-6-085 (4b), possessing a sulfonamide group (R-SO_2_NHR') in place of the *O*-tosyl (R-SO_3_R'), was prepared (Scheme 1b). It was predicted that the loss of a good leaving group would inhibit covalent modification and reduce fluorescent tagging of proteins in treated systems. Thus, as a comparison, MDA-MB-231 cell lysates were incubated separately with both 4b and 4a (100 μM) and subjected to gel electrophoresis experiments (Figure 4e). Lysates incubated with 4b showed no observable fluorescent enhancement of the protein content within the gel, while those incubated with 4a were promiscuously modified. Taken together, these results confirmed the hypothesis that the *O*-tosyl group of 4a and, by inference, S3I-201, exert their biological effect through non-specific covalent modification of cytosolic proteins.

Finally, to investigate if 4a inhibited STAT3 phosphorylation (IC_50_ ∼ 90 μM), the lysates of MDA-MB-231 cells, treated with increasing concentrations of 4a, were subjected to immunoblot analysis probing for activated pSTAT3. As Figure 4f shows, 4a recapitulated the biological effect of S3I-201, with a dose-dependent decrease in pSTAT3 levels. What is perhaps most striking though, is when the same gel is viewed under global fluorescent excitation (Figure 4f bottom panel) the observed decrease in activated pSTAT3 in response to S3I-201 is complicated by an overall increase in global protein alkylation. Thus, reductions in pSTAT3 levels might not be a result of direct and specific interactions with the STAT3 protein, but as the data collectively shows, a result of ubiquitous modification of cellular proteins.

## DISCUSSION

There are twelve cysteine residues within STAT3, Cys108, 251, 259, 328, 367, 418, 426, 468, 542, 550, 687, and 712, with different potential reactivity's to electrophiles. Cys108, not resolved in the STAT3-DNA crystal structure (PDB: 4E68), may be solvent exposed, given its proximity to the N-terminus and the conformational flexibility of that region. While Cys251, 328, and 550 are reasonable shielded, Stattic was found to modify Cys251 [27]. In the absence of a DNA binding partner, Cys468 is solvent exposed, whereas Cys367, 542, and 687 are less exposed. Of the S3I-201 modified residues, Cys108, 259, 367, 542, and 687, Cys259 was the most solvent exposed as per the crystal structure. Based on the MS/MS analysis, the remaining exposed Cys residues (418, 426, and 712) were not alkylated by S3I-201. The discrepancy in observed cysteine residues modified by Stattic (Cys251, 259, 367, and 426) as compared to S3I-201 (Cys108, 259, 367, 542, 687) is likely a result of subtle differences in reactivity. This is supported by a 2014 study by Don-Donchow *et. al.* who showed, through an MS/MS analysis, that the fungal metabolite Galiellalactone covalently modified three cysteine residues (367, 468, and 542) [28]. Interestingly, only Cys 367 was observed to be modified by Stattic, S3I-201, and Galiellalactone. Where Cys259 was modified by both Stattic and S3I-201, Cys542 was alkylated by S3I-201 and Galiellalactone. It is not clear as to whether this indicates that Cys367 is more intrinsically prone to chemical modification. Though beyond the scope of the present work, a thorough characterization of the factors affecting activity of various ‘promiscuous’ alkylators like Stattic and S3I-201 at specific sites would be a very interesting direction for future exploration.

In summary, we have demonstrated that S3I-201's *O*-tosyl serves as a leaving group, and renders S3I-201 a non-selective alkylating agent, reacting with thiol-based, as well as potentially other cellular nucleophiles via an S_N_2 mechanism. S3I-201 was also shown to react rapidly with GSH, displaying first order dependence with respect to GSH concentration and a bimolecular rate constant of 0.158 ± 0.004 h^−1^mM^−1^. As a result, S3I-201 is likely to be rapidly cleared *in vivo*. Taken together, S3I-201 will require significant structural optimization to confer a more targeted covalent effect on STAT3 over other nucleophilic residues in the cellular milieu.

## MATERIALS AND METHODS

^1^H and ^13^C NMR recorded on a Bruker 400 or Varian 500 MHz spectrometer, with solvents indicated; Chemical shifts are reported in δ (ppm). Signals were referenced to residual solvent signals or to a tetramethylsilane (TMS) internal reference. Coupling constants are reported in Hertz (Hz), integrals satisfied with respect to the expected structure, and multiplet chemical shifts measured from the approximate center of peaks. Low-resolution mass spectra were obtained on a Waters Micromass ZQ ESI mass spectrometer. Chemical reactions were monitored by thin-layer chromatography (TLC) on Merck silica gel 60 F_254_ aluminum sheets. All chemicals were purchased commercially from Sigma-Aldrich or Alfa Aesar and, unless otherwise stated, used without further purification. Column flash chromatography was performed on Agela Technologies Cleanert Silica SPE (40-60 μm average particle size and 60 Å average pore size). Preparation of 4a and 4b fluorescent probes utilized commercially available 4-amino salicylic acid, glycine tert-butyl ester hydrochloride, ethyl glycolate, and propargyl bromide as building blocks. DANSYL chloride along with 11-azido-3,6,9-trioxaundecan-1-amine were used for the construction of the fluorescent reporter/linker motifs 4a and 4b. The convergent synthesis was completed via a copper (II) sulfate-mediated azide-alkyne cycloaddition “click” reaction yielding the final fluorescent molecular probe analogues of S3I-201, 4a and 4b. The synthesis of S3I-201 and its sulfonamide derivative, DB-5-112 are provided in the Supplementary information. Unless otherwise noted STAT proteins were purchased commercially from SignalChem: STAT3 (Catalogue # S54-54G) and STAT1 (β isoform, Catalogue # S52-54G) are present as the GST-tagged version and STAT5 (Catalogue # S56-54H) as the HIS-tagged protein.

### *In vitro* chemical stability assays

Susceptibility to the biologically relevant electrophilic scavenger glutathione was tested via kinetic HPLC assays. From 10 mM compound stock solutions in DMSO 10 μL aliquots were transferred to an analytical HPLC vial containing 990 μL of reaction buffer (10.0 mM reduced GSH, 10.0 mM tris(2-carboxyethyl)phosphine hydrochloride (TCEP-HCl) and 50 mM HEPES buffered to pH 7.4) directly preceding 40.0 μL injections continued over a periodic time course. The process was automated by a Hewlett Packard Series 1100 analytical HPLC system fitted with an Agilent ZORBAX 3.5 μm Eclipse XDB-C18 column (23.0–24.0°C) under a linear elution gradient from H_2_O (double distilled and de-ionized to 18.2 MΩ cm) with 0.10 v/v % TFA to 100% HPLC grade CH_3_CN over 8.00 min followed by 2.00 min of sustained CH_3_CN under a 1.200 mL min^−1^ flow rate. Changes in the absorbance profile at 254 nm were observed and HPLC peak areas corresponding to the decay of the parent compound converting to the glutathione adduct recorded. The data, measured in three replicates was fit to a one-phase exponential decay model (GraphPad PRISM) and kinetic parameters extracted from (1).

(1) $$A(t)=\left(A_{0}-A_{\infty }\right)e^{-k_{{\text{obs}}^{t}}}+A_{\infty }$$

Where A(t) represents the HPLC peak area at time t (h), A_0_ the peak area at time zero, A∞ the extrapolated peak area at infinite time and k_obs_ the rate constant for the observed process (h^−1^). Concentration dependent reactivity studies were conducted similarly, with the exception that TCEP-HCl was excluded from the buffered system and HPLC peak areas for the decay of S3I-201 were calibrated to concentration units by application of a standard curve (Supplementary Figure S1 and Supplementary Table S1). A stock 10 mM GSH buffer solution in 100 mM HEPES pH 7.4 was diluted to the desired concentrations with 100 mM HEPES pH 7.4. Regression analysis of the obtained pseudo-first order observed rate constants, fit to (1), were analyzed by linear regression on GraphPad PRISM software to model the linear behavior of the data in accordance with the proposed chemical scheme (2) and rate law (3) to obtain the true second order rate constant (k_2_) and reaction order with respect to GSH.

(2) S3I-201+GSH→GS-S3I-201+TsOH

(3) $$\text{Rate}=k_{2}[S3I-201][\text{GSH}]$$

### Mass spectrometry

10 μg of recombinant, full-length STAT3 (SignalChem) was incubated in the presence or absence of 500 μM of S3I-201 in a 100 mM ammonium bicarbonate buffer for 1 h at 37°C. The incubation was then quenched by the addition of dithiothreitol (DTT) to a final concentration of 0.5 mM and incubation at 55°C for 30 min. Free sulfhydryl groups were alkylated with 2-iodoacetamide (Sigma) at a final concentration of 1.5 mM for 45 min at room temperature. The samples were subjected to trypsin (Promega) digestion (0.5 μg of trypsin per reaction), lyophilized, re-suspended in 0.1% formic acid, and desalted using ZipTip C18 (EMD Millipore) prior to analysis. Samples were analyzed by LC-MS/MS on a nanoLC ultra HPLC system (Eksigent) coupled to a Thermo Scientific Orbitrap-Elite.

Tryptic STAT3 peptides were resolved over an CH_3_CN gradient at a flow rate of 300 nl/min on a 15 cm long, 75-micrometer diameter C18 CL column with a particle size of 3 microns and 120 Angstrom pores (Eksigent). The aqueous and organic mobile phases consisted of 0.1% formic acid in water or CH_3_CN respectively. Organic mobile phase composition was varied over each hour long LC-MS/MS analysis as follows: 2% from 0 to 1 min, 10% to 35% from 1 to 35 min, 80% from 35.5 to 44.5 min, 2% from 45 to 60 min.

### Fluorescence spectroscopy

To ensure that replacement of the *O-*tosyl group of 4a with the sulfonamide group of 4b did not influence the overall fluorescent profile of the DANSYL fluorophore a simple fluorescence titration experiment was conducted. 40 μL of 10 mM stock DMSO solution of either 4a or 4b was diluted in 960 μL of pure CH_3_CN to give a stock 400 μM compound solution (4% DMSO). 2-fold serial dilutions were made from this stock with 4% DMSO in CH_3_CN producing a concentration range from 0.78-400 μM. 60 μL aliquots were transferred to Corning black, flat-bottom, 384-well polystyrene assay plates. Fluorescence spectral scanning of the titrations was conducted on a BioTek Cytation3 imaging reader equipped with Gen5 v2.06 imaging software using 320 nm excitation wavelengths, measuring fluorescence intensities from 350-700 nm at 5 nm step intervals. Measurements, performed in triplicate and visualized with GraphPad PRISM software (Supplementary Figure S3). Fluorescent intensities at 530 nm emission wavelengths were plotted against concentration and the linear portion of these curves fit to a standard least squares linear regression model (GraphPad PRISM) to correlate the relative fluorescence intensities of 4a and 4b to their respective concentrations within the assay.

### *In vitro* kinetic analysis of 4a with purified STAT3 protein

1 μg of purified STAT3 (SignalChem) protein was incubated with 100 μM 4a at 4°C. At the indicated time points the reaction was quenched with Laemmli loading buffer containing 1.43 M β-mercaptoethanol. Quenched samples were then subjected to 4-20% SDS-PAGE and imaged using the Chemi-Doc MP system (BIO-RAD). Following gel quantification (Image Lab software, BIO-RAD) the data points were plotted and visualized by GraphPad PRISM software.

### Gel-based STAT protein isoform labeling

1.0 μg of purified STAT3, STAT5, and STAT1 (SignalChem) were incubated with 100 uM of 4a for 16 h at 4°C. Samples were subjected to 4-20% SDS-PAGE and imaged using the Chemi-Doc MP (BIO-RAD).

### Whole cell lysates and live cell dosing

To investigate the reactivity of 4a in cell lysates, 5 × 10^6^ MDA-MB-231 cells were lysed with 200 μL RIPA buffer (Sigma-Aldrich). Lysates were treated with 0-500 μM of compound for 16 h at 4°C and subjected to 4-20% SDS-PAGE. To investigate the *in cellulo* activity 2 × 10^6^ MDA-MB-231 cells were seeded in 100 mm^2^ dishes. The following day cells were treated with 0-200 μM of 4a at 37°C, 5% CO_2_ for 6 h. Cells were lysed using 200 μL RIPA buffer (Sigma-Aldrich) and the lysates were subjected to 4-20% SDS-PAGE. Gels were visualized using the Chemi-Doc MP system. (BIO-RAD). Total protein levels were visualized using Coomassie Brilliant Blue stain. An identical protocol was performed for 4b for direct comparison with 4a.

### Immunoblotting for activated pSTAT3 expression levels in MDA-MB-231 cells

Lysates were reduced, boiled, subjected to 4-20% SDS-PAGE and transferred to LF-PVDF membranes. Membranes were blocked with 3% BSA and incubated with pSTAT3 (Abcam ab76315), STAT3 (Cell Signaling 4904) or B-Actin (Cell signaling 3700) at 4°C overnight. Membranes were then probed with either anti-mouse IgG Alexa Flour 488 (Cell Signaling 4408) or anti-rabbit IgG Alexa Fluor 647 (Cell Signaling 4414) for 1 h at room temperature. Membranes were visualized using the Chemi-Doc MP System (BIO-RAD).

### Supplementary information

Detailed chemical synthesis protocols, calibration curve relating HPLC peak areas to S3I-201 concentrations (Supplementary Figure S1, Supplementary Table S1), data tables for kinetic curve fitting and extracted parameters (Supplementary Table S2, S3, S4 and S5), fluorescence titration experiments with 4a and 4b (Supplementary Figure S3), LC-MS/MS analysis of peptide fragment showing modification of Cys108 and expanded data tables for MS/MS fragment ions of all sequences (Supplementary Figure S2 and Supplementary Tables S6, S7, S8, S9, S10).

## SUPPLEMENTARY FIGURES AND TABLES


