# Supplementary material for: Signal transducer and activator of transcription 3 (STAT3) inhibitor, S3I-201, acts as a potent and non-selective alkylating agent
Source: Oncotarget. 2016 Mar 2;7(15):20669–79. doi: 10.18632/oncotarget.7838 (PMC4991483; doi:10.18632/oncotarget.7838)
Supplement: Supplementary file 1 [file oncotarget-07-20669-s001.pdf]

# CHEMICAL SYNTHESIS S3I-201 (NSC 74859)

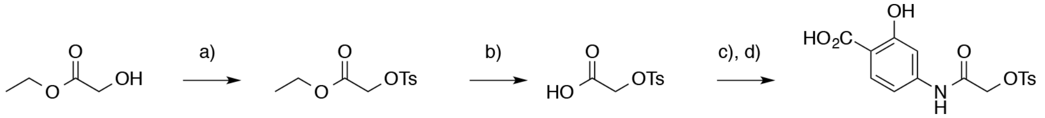

**Scheme 1: a.** TsCl, $Et_3N$, $CH_3CN$, 0°C - rt, 75%; **b.** 5% aq. NaOH/EtOH (2:1), 97.6%; **c.** $SOCl_2$, reflux, 2 h; **d.** i) 4-amino salicylic acid, THF, $Na_2CO_3$, ii) Acid chloride in THF, iii) DIPEA/$H_2O$, 68.9% over 2 steps.

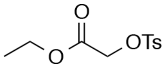

## Ethyl 2-(tosyloxy)acetate

909 µL of ethyl glycolate (9.6 mmol) was dissolved in 96 mL acetonitrile over an ice bath at 0°C. To the stirring solution was added 1.6 mL of triethylamine (11.5 mmol) followed by 2.20 g of p-toluene sulfonyl chloride (11.5 mmol). The reaction gradually equilibrated to room temperature and monitored by thin-layer chromatography (25% EtOAc in hexanes), visualized by $KMnO_4$ staining. The reaction was judged complete by exhaustion of ethyl glycolate ($R_f$ = 0.23). The acetonitrile was removed *in vacuo,* the residue diluted with EtOAc and poured over $H_2O$. The aqueous layer was extracted with 75 mL portions of EtOAc (x2) and the combined organics washed with 100 mL of $H_2O$ (x2), 100 mL saturated NaCl (x1) and dried over anhydrous $Na_2SO_4$. Excess solvent was removed *in vacuo* to produce loose yellow oil subsequently adsorbed onto silica gel for flash chromatography (mobile phase: 20% EtOAc in hexanes). Evaporation of eluent for $R_f$ = 0.43 (25% EtOAc in hexanes) gave 1.86 g of yellow oil that solidified on cooling to room temperature. Confirmed by $^1H$, $^{13}C$ NMR and LRMS analysis (75.0% yield). $^1H$ NMR (400 MHz, Chloroform-*d*) δ 1.23 (t, $J$ = 7.1 Hz, 3H), 2.44 (s, 3H), 4.17 (q, $J$ = 7.1 Hz, 2H), 4.57 (s, 2H), 7.35 (d, $J$ = 8.0 Hz, 2H), 7.82 (d, $J$ = 8.3 Hz, 2H); $^{13}C$ NMR (101 MHz, $CDCl_3$) δ 14.1, 21.8, 62.0, 64.8, 128.2, 130.0, 132.8, 145.4, 166.1; LRMS, *m/z* +ESI calcd for $[C_{11}H_{14}O_5S + H]^+$, 259.30 – found 280.76 [M + Na].

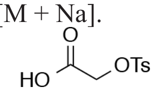

## 2-(tosyloxy)acetic acid

1.28 g (4.9 mmol) of pale yellow ethyl ester was suspended in 6.0 mL of a 2:1 mix of EtOH/5% aq. NaOH under gentle heating that, when dissolved, was allowed to stir at room temperature. The reaction was assessed via thin layer chromatography (92% $CH_2Cl_2$, 7% $CH_3OH$, 1% AcOH) and judged complete by the disappearance of the ethyl ester ($R_f$ = 0.89). Excess EtOH was removed *in vacuo* and the solution poured over 5% aq. HCl and extracted with 100 mL portions of EtOAc (x2). The combined organic layers were washed with 75 mL portions of 5% aq. HCl (x2), saturated NaCl (x1) and dried over anhydrous $Na_2SO_4$. Excess solvent was removed *in vacuo* to give 1.06 g of bright white solid powder with no further purification required. Confirmed by $^1H$, $^{13}C$ NMR and LRMS analysis (93% yield). $^1H$ NMR (400 MHz, Methanol-$d_4$) δ 2.45 (s, 3H), 4.59 (s, 2H), 7.44 (d, $J$ = 8.1 Hz, 2H), 7.82 (d, $J$ = 8.2 Hz, 2H); $^{13}C$ NMR (101 MHz, MeOD) δ 21.6, 49.0, 65.9, 129.1, 131.1, 134.1, 146.8, 169.4; LRMS, *m/z* -ESI calcd for $[C_9H_{10}O_5S - H]^-$, 229.23 - mass not detected.

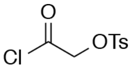

## 2-chloro-2-oxoethyl 4-methylbenzenesulfonate

170 mg (0.74 mmol) of the free acid was dissolved in 1.5 mL of thionyl chloride over an oil bath heated to 80 °C and allowed to reflux for 2 h. Excess thionyl chloride was removed *in vacuo* with a saturated $NaHCO_3$ base trap to give a slightly brown white solid that was carried forward without further purification or characterization.

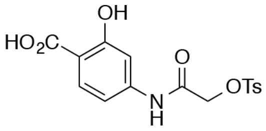

## 2-hydroxy-4-(2-(tosyloxy)acetamido)benzoic acid

113 mg (0.74 mmol) of 4-amino salicylic acid was mixed with 91 mg (0.74 mmol) of $Na_2CO_3$. This mixture was then taken up in 1.5 mL of THF to give a stirring suspension held under an atmosphere of $N_2$. Separately, the acid chloride was dissolved in 1.5 mL

of THF and introduced directly to the stirring solution, giving a brown suspension. After several hours 128 µL (0.74 mmol) of DIPEA was added, followed by $H_2O$ drop wise to clear the suspension. The solution was then poured over 50 mL of 1.0 M HCl and extracted with 25 mL portions of EtOAc (x3). The combined organics were washed with 25 mL portions of 1.0 M HCl (x2), dried over anhydrous $Na_2SO_4$, filtered and evaporated *in vacuo* to give 186 mg of light brown solid (68.9% crude yield). 86 mg was taken up in $CH_3CN$ (with 4 drops DMSO) and purified by reverse phase HPLC and lyophilized to give 38 mg of white solid powder. $^1H$ NMR (400 MHz, DMSO-$d_6$) δ 2.40 (s, 3H), 4.70 (s, 2H), 6.98 (dd, $J$ = 8.7, 1.8 Hz, 1H), 7.23 (d, $J$ = 1.7 Hz, 1H), 7.49 (d, $J$ = 8.2 Hz, 2H), 7.72 (d, $J$ = 8.7 Hz, 1H), 7.83 (d, $J$ = 8.2 Hz, 2H), 10.36 (s, 1H), 11.35 (s, 1H); $^{13}C$ NMR (101 MHz, DMSO) δ 21.1, 67.3, 106.3, 108.3, 110.4, 127.8, 130.2, 131.1, 132.0, 144.3, 145.4, 161.9, 163.9, 171.5; LRMS, *m/z* +ESI calcd for $[C_{16}H_{15}NO_7S + H]^+$, 366.06 - found 366.08; LRMS, *m/z*–ESI calcd $[C_{16}H_{15}NO_7S – H]^-$, 364.05 - found 364.04; 68.9% crude yield, 14% isolated yield.

## CHEMICAL SYNTHESIS OF SULFONAMIDE DB-5-112

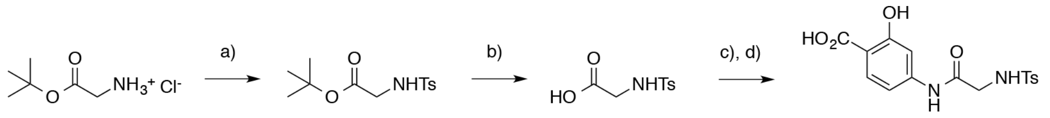


**Scheme 2: a.** TsCl, $Et_3N$, $CH_3CN$, 0°C - rt, 90.8%; **b.** TFA/$CH_2Cl_2$ (1:2), 30 min, 51.9%; **c.** $SOCl_2$, reflux, 2 h; **d.** i) 4-amino salicylic acid, THF, $Na_2CO_3$, ii) Acid chloride in THF, iii) DIPEA/$H_2O$, 48.8% over 2 steps.

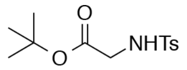


### *Tert*-butyl tosylglycine

750 mg (4.5 mmol) of glycine *tert*-butyl ester hydrochloride was dissolved in 40.0 mL $CH_3CN$ to give a stirring clear, colorless solution. To this, was added 2.0 mL (14.8 mmol) triethylamine prompting a white solid to separate from solution. Separately, 938 mg (4.9 mmol) *p*-toluene sulfonyl chloride was dissolved in 5.0 mL $CH_3CN$ and added drop wise to the stirring suspension. The mixture was allowed to stir for 3 h observing the formation of product ($R_f$ = 0.41, 25% EtOAc in hexanes) by thin-layer chromatography of the reaction mixture, visualized with $KMNO_4$ staining. Excess $CH_3CN$ solvent was evaporated *in vacuo*, taken up in EtOAc and poured over 50 mL $H_2O$ in a separatory funnel. This solution was extracted with 50 mL portions of EtOAc (x3), washed with 50 mL portions of $H_2O$ (x2), 50 mL sat. NaCl (x1) and dried over anhydrous $Na_2SO_4$. On evaporation *in vacuo* the residue was adsorbed onto silica and columned isocratically in 25% EtOAc in hexanes to isolate the product as 1.21 g of white solid. Confirmed by $^1H$, $^{13}C$ NMR and LRMS analysis (94.5% yield). $^1H$ NMR (400 MHz, Chloroform-*d*) δ 1.32 (s, 9H), 2.40 (s, 3H), 3.65 (d, $J$ = 5.5 Hz, 2H), 5.10 (t, $J$ = 5.1 Hz, 1H), 7.29 (d, $J$ = 8.1 Hz, 2H), 7.73 (d, $J$ = 8.2 Hz, 2H); $^{13}C$ NMR (101 MHz, $CDCl_3$) δ 21.4, 27.7, 44.6, 82.7, 127.2, 129.6, 136.1, 143.5, 167.6; LRMS, *m/z* +ESI calcd for $[C_{13}H_{19}NO_4S + H]^+$, 286.11 - found: 308.01 (M + Na), 324.13 (M + K).

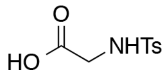


### Tosylglycine

1.05 g (3.7 mmol) of the *tert*-butyl ester sulfonamide was dissolved in 10.0 mL $CH_2Cl_2$. To this was added 5.0 mL of TFA. The solution was allowed to stir at room temperature for 30 min and judged complete by depletion of $R_f$ = 0.76 (50% EtOAc in hexanes, $KMnO_4$ visualization) starting material with formation of $R_f$ = 0.30 spot. Excess $CH_2Cl_2$ was evaporated *in vacuo* and the residue triturated with $Et_2O$ to give 577 mg of pure white solid. Confirmed by $^1H$, $^{13}C$ NMR (68.2% yield). $^1H$ NMR (400 MHz, DMSO-$d_6$) δ 2.37 (s, 3H), 3.55 (d, $J$ = 6.1 Hz, 2H), 7.36 (d, $J$ = 8.0 Hz, 2H), 7.67 (d, $J$ = 8.1 Hz, 2H), 7.94 (t, $J$ = 6.1 Hz, 1H), 12.67 (s, 1H); $^{13}C$ NMR (101 MHz, DMSO) δ 21.3, 44.1, 126.8, 129.8, 138.1, 142.9, 170.5; LRMS, *m/z* +ESI calcd for $[C_9H_{11}NO_4S + H]^+$, 230.05 - found 252.05.

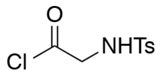


### Tosylglycine chloride

88 mg (0.4 mmol) of the free acid was dissolved in 2.0 mL of thionyl chloride over an oil bath heated to 80 °C and allowed to reflux for 2 h. Excess thionyl chloride was removed *in vacuo* with a saturated $NaHCO_3$ base

trap to give a slightly brown white solid that was carried forward to the next reaction without further purification or characterization.

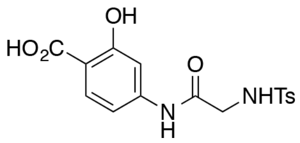


**2-hydroxy-4-(2(4-methylphenyl)sulfonamido) acetamido)benzoic acid, d)**

58 mg (0.4 mmol) of 4-amino salicylic acid was mixed with 47 mg (0.4 mmol) of $Na_2CO_3$. This mixture was then taken up in 1.5 mL of THF to give a stirring suspension held under an atmosphere of $N_2$. Separately, the acid chloride was dissolved in 1.5 mL of THF and introduced directly to the stirring solution, producing a cloudy, yellow-orange suspension. After several hours 66 µL (0.4 mmol) of DIPEA was added, followed by $H_2O$ drop wise to clear the suspension to a brown solution. The solution was then poured over 50 mL of 1.0 M HCl and extracted with 25 mL portions of EtOAc (x4). The combined organics were washed with 25 mL portions of 1.0 M HCl (x2), a 25 mL portion of saturated NaCl, dried over anhydrous $Na_2SO_4$, filtered and evaporated *in vacuo* to give an orange brown residue. This residue was purified by reverse phase HPLC and lyophilized to give 68 mg of off-white solid powder. Confirmed by $^{1}$H, $^{13}$C NMR and LRMS analysis (48.8% isolated yield). $^{1}$H NMR (400 MHz, DMSO-$d_6$) δ 2.35 (s, 3H), 3.66 (d, $J$ = 6.1 Hz, 2H), 6.95 (dd, $J$ = 8.7, 1.9 Hz, 1H), 7.23 (d, $J$ = 1.9 Hz, 1H), 7.36 (d, $J$ = 8.2 Hz, 2H), 7.70 (d, $J$ = 8.2 Hz, 3H), 8.01 (t, $J$ = 6.2 Hz, 1H), 10.15 (s, 1H), 11.34 (s, 1H); $^{13}$C NMR (101 MHz, DMSO) δ 20.9, 45.9, 106.0, 107.8, 110.2, 126.6, 129.5, 131.0, 137.5, 142.7, 144.9, 162.0, 167.0, 171.5; LRMS, *m/z* +ESI for $[C_{16}H_{16}N_2O_6S + H]^+$, calcd 365.08 - found 365.17.

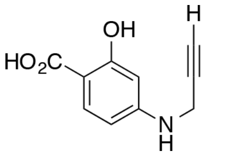


**2-hydroxy-4-(prop-2-yn-1-ylamino)benzoic acid, (1)**

1.83 g (11.9 mmol) of 4-amino salicylic acid was dissolved in 50 mL DMF in a 500 mL round-bottom flask, placed over an ice-bath, fitted with a 250 mL addition funnel. To this stirring solution was added 3.89 g (11.9 mmol) caesium carbonate. The propargyl bromide, diluted in 100 mL DMF was transferred to the addition funnel, followed by drop wise addition to the stirring solution. A remaining 50 mL of DMF was required to wash the contents from the funnel and the solution allowed to equilibrate to room temperature. The reaction progress was tracked by thin layer chromatography (98% $CH_2Cl_2$, 1.75% $CH_3OH$, 0.25% $CH_3CO_2H$) by the disappearance of 4-amino salicylic acid starting material ($R_f$ = 0.09) and concomitant formation of the product spot ($R_f$ = 0.69). The reaction contents were then poured over 100 mL 1.0 M HCl and extracted with 75 mL portions of EtOAc (x4). The combined organics were then washed with 1.0 M HCl (x3), saturated NaCl (x1) and dried over anhydrous $Na_2SO_4$ and the solvent removed *in vacuo*. The residue was then adsorbed onto silica and columned isocratically in 98.4% $CH_2Cl_2$, 1.4% $CH_3OH$, 0.2% $CH_3CO_2H$ to isolate the identified $R_f$ = 0.69 spot cleanly as 1.524 g of pale yellow solid. Confirmed by $^{1}$H, $^{13}$C NMR and LRMS analysis (83.6% yield). $^{1}$H NMR (400 MHz, DMSO-$d_6$) δ 3.53 (s, 1H), 4.85 (s, 2H), 5.98 (s, 1H), 6.11 (d, $J$ = 8.8 Hz, 1H), 6.17 (s, 2H), 7.42 (d, $J$ = 8.8 Hz, 1H), 10.51 (s, 1H); $^{13}$C NMR (101 MHz, DMSO) δ 51.8, 77.8, 78.6, 98.6, 99.0, 106.8, 131.2, 156.4, 163.0, 168.4; LRMS, *m/z* -ESI for $[C_{10}H_9NO_3 - H]^-$, calcd: 190.05 – found 189.94.

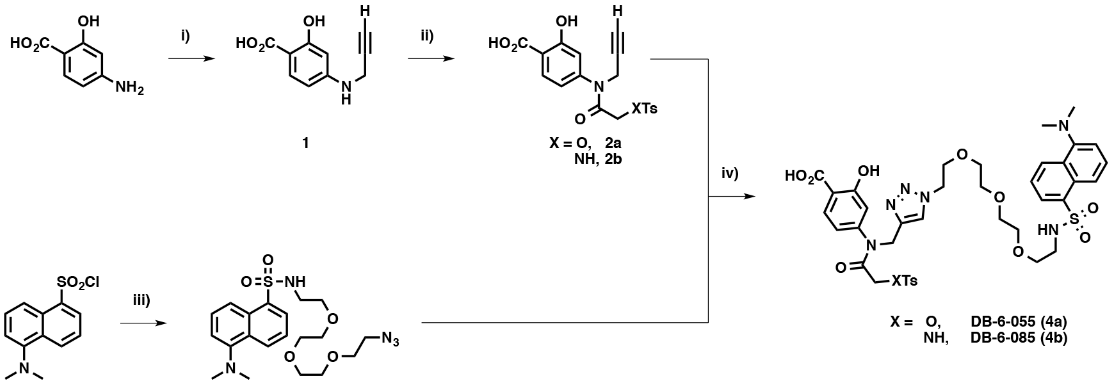


**Scheme 3: i.** $Cs_2CO_3$, 80 wt% propargyl bromide, DMF, 0°C – rt; **ii.** 1) $Na_2CO_3$, THF; 2) 2-Chloro-2-oxoethyl 4-methylbenzenesulfonate or tosylglycine, THF; 3) DIPEA/$H_2O$; **iii.** 11-azido-3,6,9-trioxaundecane-1-amine, $CH_2Cl_2$, DIPEA, 0°C – rt; **iv.** $CuSO_4$, sodium ascorbate, $H_2O$/THF, rt.

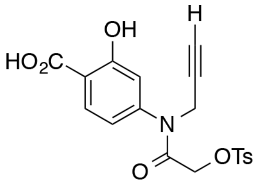


**2-hydroxy-4-(*N*-(prop-2-yn-1-yl)-2-(tosyloxy) acetamido)benzoic acid, (2a)**

124 mg (0.5 mmol) of 2-(tosyloxy)acetic acid was dissolved in 5.0 mL of thionyl chloride over an oil bath heated to 80°C and allowed to reflux for 2 h. Excess thionyl chloride was removed *in vacuo* with a saturated $NaHCO_3$ base trap to give a slightly brown white solid acid chloride that was carried forward to the next reaction without further purification or characterization. Separately, 103 mg (0.5 mmol) of N-alkynylated salicylic acid was dissolved with 5 mL THF and 67 mg (0.5 mmol) $Na_2CO_3$ added, along with the acid chloride (dissolved in 1.0 mL THF) to give a stirring yellow suspension. After several hours 94 µL(0.5 mmol) DIPEA was added, followed by $H_2O$ drop wise to clear the suspension. The reaction was judged complete by thin layer chromatography ($R_f$ = 0.68, 99.6% $CH_2Cl_2$, 0.35% $CH_3OH$, 0.05% $CH_3CO_2H$). The solution was then poured over 50 mL 1.0 M HCl and extracted with 50 mL portions of EtOAc (x2). The combined organic layers were then washed with 25 mL portions of 1.0 M HCl (x1), saturated NaCl (x1), and dried over anhydrous $Na_2SO_4$ to obtain a brown, viscous oil after evaporation *in vacuo*. The residue was adsorbed onto silica and columned isocratically in 99.2% $CH_2Cl_2$, 0.70% $CH_3OH$, 0.10% $CH_3CO_2H$ to isolate $R_f$ = 0.68 spot as 173.0 mg of fluffy white solid. Confirmed by $^1H$, $^{13}C$ NMR (79.4% yield). $^1H$ NMR (400 MHz, DMSO-$d_6$) δ 2.40 (s, 3H), 3.64 (t, $J$ = 2.0 Hz, 1H), 4.71 (s, 2H), 4.95 (d, $J$ = 2.1 Hz, 2H), 7.00 (dd, $J$ = 8.9, 1.5 Hz, 1H), 7.30 (d, $J$ = 1.3 Hz, 1H), 7.49 (d, $J$ = 8.1 Hz, 2H), 7.73 (d, $J$ = 8.7 Hz, 1H), 7.84 (d, $J$ = 8.1 Hz, 2H), 10.40 (s, 1H), 10.43 (s, 1H); $^{13}C$ NMR (101 MHz, DMSO) δ 21.1, 52.5, 67.3, 78.2, 78.2, 106.6, 108.0, 110.6, 127.8, 130.2, 131.1, 132.0, 144.5, 145.4, 161.0, 164.0, 167.1; LRMS, *m/z*+ESI for $[C_{19}H_{17}NO_7S + H]^+$, calcd: 404.08 – found 404.13 (M + H), 426.13 (M + Na); LRMS, *m/z* -ESI for $[C_{19}H_{17}NO_3S–H]^-$, calcd: 402.07 – found 402.15.

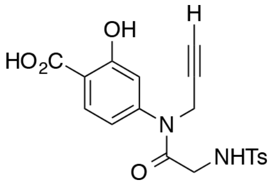


**2-hydroxy-4-(2-((4-methylphenyl)sulfonamide)-*N*-(prop-2-yn-1-yl)acetamido)benzoic acid, (2b)**

454 mg (2.0 mmol) of the free acid (made previously in synthesis **2b**) was dissolved in 5.0 mL of thionyl chloride over an oil bath heated to 80°C and allowed to reflux for 2 h. Excess thionyl chloride was removed *in vacuo* with a saturated $NaHCO_3$ base trap to give a slightly brown white solid acid chloride that was carried forward to the next reaction without further purification or characterization. Separately, 388 mg (2.0 mmol) of N-alkynylated salicylic acid was dissolved with 15 mL THF and 246 mg (2.0 mmol) $Na_2CO_3$ added, along with the acid chloride (dissolved in 3.0 mL THF) to give a stirring yellow suspension. After several hours 690 µL (4.0 mmol) DIPEA was added, followed by $H_2O$ drop wise to clear the suspension. The reaction was judged complete by thin layer chromatography ($R_f$ = 0.19, 98% $CH_2Cl_2$, 1.75% $CH_3OH$, 0.25% $CH_3CO_2H$). The solution was then poured over 75 mL 1.0 M HCl and extracted with 50 mL portions of EtOAc (x3). The combined organic layers were then washed with 50 mL portions of 1.0 M HCl (x2), saturated NaCl (x1) and dried over anhydrous $Na_2SO_4$ to obtain a yellow residue after evaporation *in vacuo*. The residue was adsorbed onto silica and columned isocratically in 98% $CH_2Cl_2$, 1.75% $CH_3OH$, 0.25% $CH_3CO_2H$ to isolate the $R_f$ = 0.19 spot as 538 mg of light brown solid. Confirmed by $^1H$, $^{13}C$ NMR and LRMS (67.5% yield). $^1H$ NMR (400 MHz, DMSO-$d_6$) δ 2.35 (s, 3H), 3.64 (d, $J$ = 1.9 Hz, 1H), 3.67 (d, $J$ = 5.8 Hz, 2H), 4.95 (s, 2H), 6.98 (d, $J$ = 8.8 Hz, 1H), 7.31 (s, 1H), 7.36 (d, $J$ = 7.8 Hz, 2H), 7.67 - 7.74 (m, 3H), 8.02 (t, $J$ = 5.8 Hz, 1H), 10.20 (s, 1H), 10.42 (s, 1H); $^{13}C$ NMR (101 MHz, DMSO) δ 20.9, 46.0, 52.5, 78.2, 106.3, 107.4, 110.5, 126.6, 129.5, 131.0, 137.5, 142.7, 145.1, 161.1, 167.1, 167.3; LRMS, *m/z* +ESI for $[C_{19}H_{18}N_2O_6S + H]^+$, calcd: 403.10 – found 403.18 (M + H), 425.11 (M + Na), 440.97 (M + K).

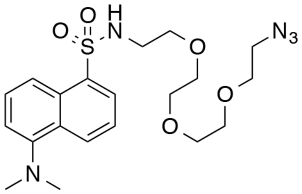


***N*-(2-(2-(2-(2-azidoethoxy)ethoxy)ethoxy)ethyl)-5-(dimethylamino)naphthalene-1-sulfonamide, (3)**

291 mg (1.1 mmol) Dansyl chloride was dissolved in 5.0 mL $CH_2Cl_2$ in a 20 mL reaction vial wrapped in Aluminum foil, over an ice bath followed by the addition of 207 µL (1.2 mmol) DIPEA. To the stirring solution was added 283 mg (1.3 mmol) 11-azido-3,6,9-trioxaundecane-1-amine pre-dissolved in 5.0 mL $CH_2Cl_2$ drop wise. The solution was allowed to stir at room temperature for 10 hours and visualized by thin layer chromatography (50%

EtOAc in heptane) to observe the depletion of dansyl chloride ($R_f$ = 0.63) and formation of new, fluorescent product spot ($R_f$ = 0.18). The excess $CH_2Cl_2$ was removed *in vacuo* and the residue adsorbed directly onto silica for flash chromatography in 40% heptane in EtOAc to isolate the $R_f$ = 0.18 spot as 431 mg of translucent green oil. Confirmed by $^1$H, $^{13}$C NMR and LRMS analysis (88.3% yield). $^1$H NMR (400 MHz, Chloroform-*d*) δ 2.87 (s, 6H), 3.09 (q, *J* = 5.2 Hz, 2H), 3.36 (t, *J* = 4.6 Hz, 6H), 3.46 - 3.52 (m, 2H), 3.57 - 3.62 (m, 2H), 3.62 - 3.69 (m, 4H), 5.50 (t, *J* = 5.7 Hz, 1H), 7.17 (d, *J* = 7.5 Hz, 1H), 7.50 (t, *J* = 7.9 Hz, 1H), 7.55 (t, *J* = 8.1 Hz, 1H), 8.23 (d, *J* = 7.3 Hz, 1H), 8.30 (d, *J* = 8.7 Hz, 1H), 8.52 (d, *J* = 8.5 Hz, 1H); $^{13}$C NMR (101 MHz, $CDCl_3$) δ 34.3, 43.2, 45.5, 50.7, 69.3, 70.1, 70.3, 70.5, 70.7, 70.7, 115.3, 119.0, 123.3, 128.4, 129.4, 129.7, 123.0, 130.4, 135.2, 152.0; LRMS, *m/z* +ESI for $[C_{20}H_{29}N_5O_5S + H]^+$, calcd: 452.20 – found 452.20.

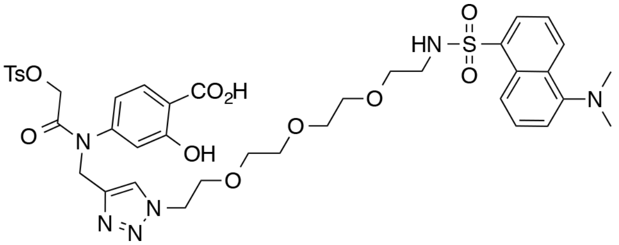


**4-(*N*-((1-(2-(2-(2-(2-((5-(dimethylamino)naphthalene)-1-sulfonamido)ethoxy)ethoxy)ethoxy)ethyl)-1*H*-1,2,3-triazol-4-yl)methyl)-2-(tosyloxy)acetamido)-2-hydroxybenzoic acid (4a)**

75 mg (0.2 mmol) of the fluorescent azide (*N*-(2-(2-(2-(2-azidoethoxy)ethoxy)ethoxy)ethyl)-5-(dimethylamino)naphthalene-1-sulfonamide) was measure and dissolved in 330 µL THF in a 2.0 mL reaction vial wrapped in Aluminum foil followed by 67 mg (0.2 mmol) of the tosylated alkyne transferred to the stirring solution. Separately, 6.5 mg (0.03 mmol) sodium ascorbate was dissolved in 330 µL $H_2O$ and introduced to the reaction mixture. Finally, 0.5 mg (0.003 mmol) copper (II) sulfate was added. The reaction was capped, protected from light and allowed to react overnight. Thin layer chromatography (10% $CH_3OH$ in $CH_2Cl_2$) revealed the depletion of the alkyne starting material ($R_f$ = 0.92), the fluorescent azide ($R_f$ = 0.88) and the formation of an intensely fluorescent new spot ($R_f$ = 0.76). The reaction was diluted with EtOAc, poured over 25.0 mL $H_2O$ and extracted with 20.0 mL portions of EtOAc (x3). The combined organics were washed with $H_2O$ (x3), saturated NaCl (x1) followed by drying over anhydrous $Na_2SO_4$. Evaporation *in vacuo* gave 166 mg of crude green oil that was purified by reverse phase HPLC and lyophilized to 62 mg of slightly green white powder. Confirmed by $^1$H, $^{13}$C, HSQC, HMBC, TOCSY NMR and LRMS analysis. (44.2% isolated yield). $^1$H NMR (500 MHz, DMSO-$d_6$) δ 2.37 (s, 3H), 2.83 (s, 6H), 2.93 (q, *J* = 5.8 Hz, 2H), 3.18 – 3.29 (m, 6H), 3.36 (dd, *J* = 5.7, 3.7 Hz, 2H), 3.45 (dd, *J* = 5.8, 3.6 Hz, 2H), 3.78 (t, *J* = 5.2 Hz, 2H), 4.51 (t, *J* = 5.2 Hz, 2H), 4.69 (s, 2H), 5.38 (s, 2H), 6.96 (dd, *J* = 8.8, 2.0 Hz, 1H), 7.26 – 7.30 (m, 2H), 7.46 (d, *J* = 8.0 Hz, 2H), 7.55 – 7.59 (m, 1H), 7.59 – 7.62 (m, 1H), 7.68 (s, 1H), 7.81 (d, *J* = 8.3 Hz, 2H), 7.98 (t, *J* = 5.8 Hz, 1H), 8.10 (dd, *J* = 7.3, 1.0 Hz, 1H), 8.18 (s, 1H), 8.31 (d, *J* = 8.7 Hz, 1H), 8.43 (d, *J* = 8.5 Hz, 1H), 10.38 (s, 1H), 10.55 (s, 1H); $^{13}$C NMR (101 MHz, DMSO) δ 20.7, 21.0, 40.1, 42.2, 45.1, 49.4, 58.0, 67.2, 68.5, 68.9, 69.4, 69.4, 69.4, 69.5, 106.5, 107.9, 110.6, 115.2, 119.5, 123.6, 125.3, 125.4, 127.7, 127.8, 128.0, 128.0, 128.9, 129.0, 129.1, 130.1, 130.9, 131.9, 136.3, 137.6, 141.3, 144.4, 145.3, 145.6, 150.8, 161.1, 163.9, 167.8; LRMS, *m/z* +ESI for $[C_{39}H_{46}N_6O_{12}S_2 + H]^+$, calcd 855.27 - detected: 855.26 (M + H), 877.26 (M + Na).

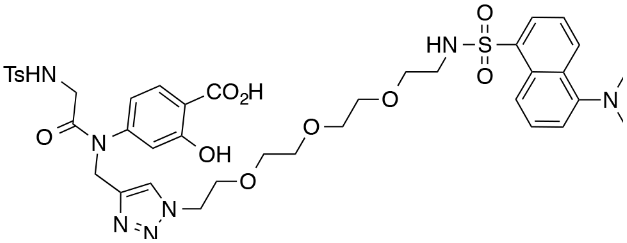


**4-(*N*-((1-(2-(2-(2-(2-((5-(dimethylamino)naphthalene)-1-sulfonamido)ethoxy)ethoxy)ethoxy)ethyl)-1*H*-1,2,3-triazole-4-yl)methyl)-2-((4-methylphenyl)sulfonamide)acetamido)-2-hydroxybenzoic acid (4b)**

83 mg (0.2 mmol) of the fluorescent azide (*N*-(2-(2-(2-(2-azidoethoxy)ethoxy)ethoxy)ethyl)-5-(dimethylamino)naphthalene-1-sulfonamide) was measure and dissolved in 330 µL THF in a 2.0 mL reaction vial wrapped in Aluminum foil followed by 74 mg (0.2 mmol) of the sulfonamide alkyne transferred to the stirring solution. Separately, 3.7 mg (0.02 mmol) sodium ascorbate was dissolved in 330 µL $H_2O$ and introduced to the reaction mixture. Finally, 0.3 mg (0.002 mmol) copper (II) sulfate was added. The reaction was capped, protected from light and allowed to react overnight. Thin layer chromatography (10% $CH_3OH$ in $CH_2Cl_2$) revealed the depletion of the alkyne starting material ($R_f$ = 0.62), the fluorescent azide ($R_f$ = 0.73) and the formation of an intensely fluorescent new spot ($R_f$ = 0.53). The reaction was diluted with EtOAc, poured over 25.0 mL $H_2O$ and extracted with 20 mL portions of EtOAc (x3). The combined organics were washed with $H_2O$ (x2), saturated NaCl (x1) followed by drying over anhydrous $Na_2SO_4$. Evaporation *in vacuo* gave crude green oil that was purified by reverse phase HPLC and lyophilized to 90 mg of slightly green white powder. Confirmed by $^1$H, $^{13}$C, HSQC, HMBC, TOCSY NMR and LRMS analysis. (57.0% isolated yield). $^1$H NMR (500

MHz, DMSO-$d_6$) δ 2.32 (s, 3H), 2.82 (s, 6H), 2.92 (q, *J* = 5.8 Hz, 2H), 3.19 – 3.28 (m, 6H), 3.33 – 3.38 (m, 2H), 3.42 – 3.47 (m, 2H), 3.64 (d, *J* = 5.9 Hz, 2H), 3.77 (t, *J* = 5.3 Hz, 2H), 4.50 (t, *J* = 5.2 Hz, 2H), 5.37 (s, 2H), 6.93 (dd, *J* = 8.8, 2.1 Hz, 1H), 7.26 (d, *J* = 7.4 Hz, 1H), 7.27 (d, *J* = 2.0 Hz, 1H), 7.34 (dd, *J* = 9.3, 0.7 Hz, 2H), 7.56 (dd, *J* = 8.7, 7.5 Hz, 1H), 7.59 (dd, *J* = 8.7, 7.3 Hz, 1H), 7.65 (d, *J* = 8.8 Hz, 1H), 7.67 (dt, *J* = 8.2, 2.0 Hz, 2H), 7.98 (t, *J* = 5.8 Hz, 2H), 8.09 (dd, *J* = 7.3, 1.2 Hz, 1H), 8.17 (s, 1H), 8.29 (d, *J* = 8.7 Hz, 1H), 8.43 (dt, *J* = 8.5, 1.0 Hz, 1H), 10.18 (s, 1H), 10.54 (s, 1H); $^{13}$C NMR (126 MHz, dmso) δ 21.4, 42.7, 45.6, 46.4, 49.9, 58.5, 69.0, 69.4, 69.9, 69.9, 69.9, 70.0, 106.7, 107.9, 110.9, 115.7, 120.1, 124.1, 125.8, 127.1, 128.2, 128.5, 129.3, 129.5, 129.6, 130.0, 131.3, 136.8, 137.9, 141.8, 143.2, 145.5, 161.7, 167.6, 168.5; LRMS, *m/z* +ESI for $[C_{39}H_{47}N_7O_{11}S_2 + H]^+$, calcd 854.28 - detected: 854.30 (M + H), 876.23 (M + Na).

## SUPPLEMENTARY FIGURES AND TABLES

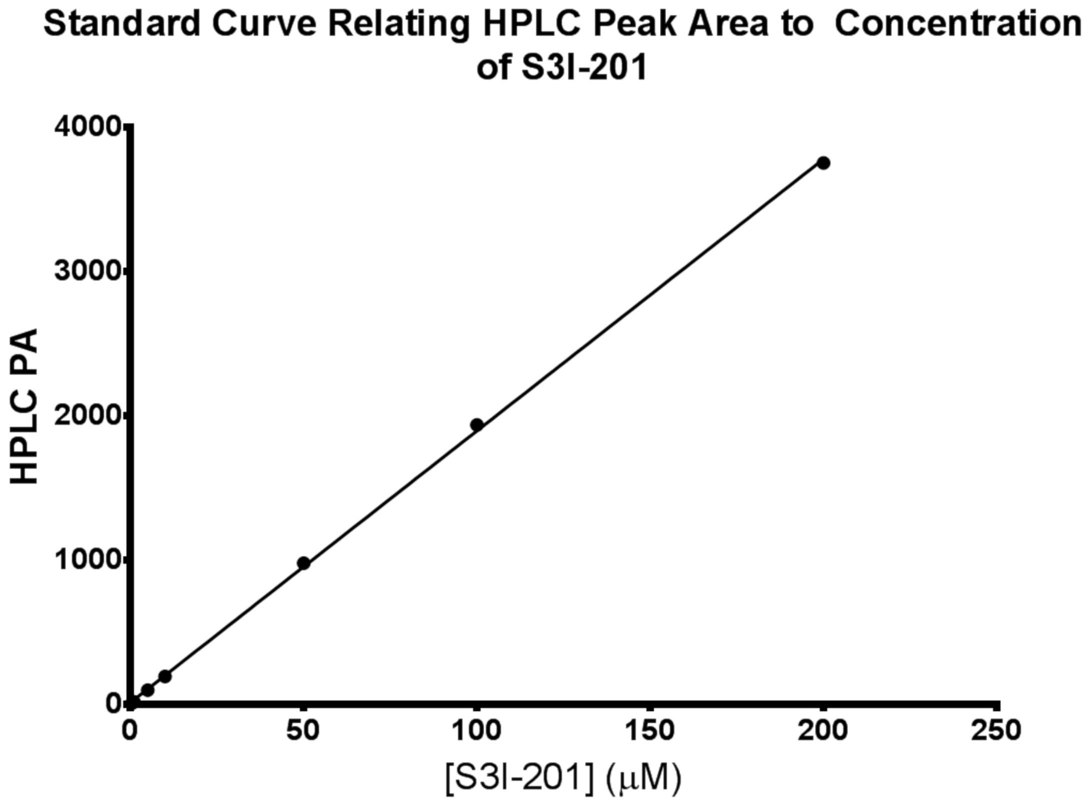


**Supplementary Figure S1: Analytical HPLC calibration curve relating measured HPLC peak areas to the concentration of [S3I-201] in solution.** Briefly, 200, 100, 50, 10, 5, 1, 0.5 μM concentrations of S3I-201 were prepared from a 10 mM stock solution (in DMSO) to a final volume of 1.0 mL in Supelco low absorption, 2 mL, clear glass, analytical HPLC vials capped with PTFE/silicone septa, using a 100 mM HEPES solution buffered to pH 7.4. Sample dilutions were created individually to avoid serial dilution errors and measured with 40 μL injections over 3 replicates, consistent with all previous experimental procedures. Data points are expressed as the mean value ± standard error.

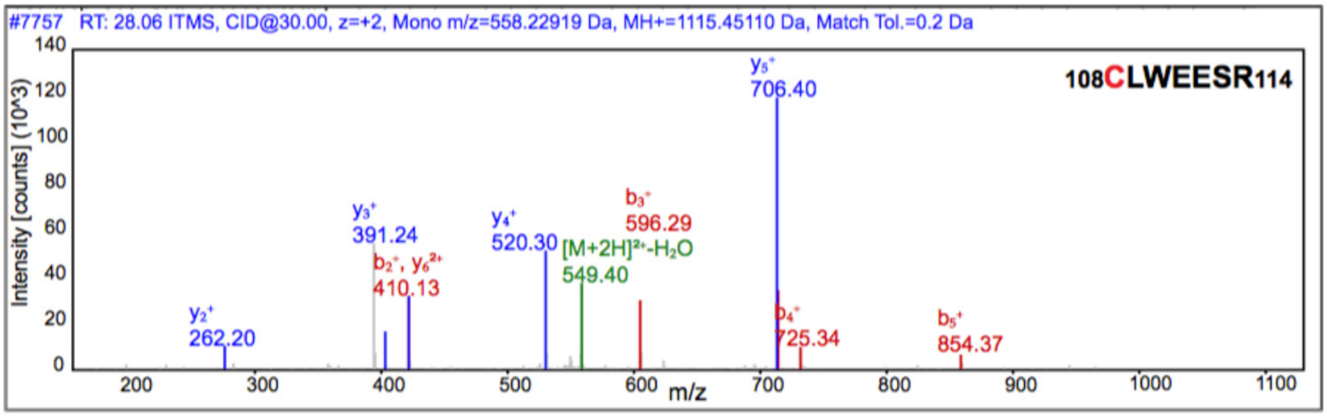


**Supplementary Figure S2: LC-MS/MS fragment analysis of the 108CLWEESR114 peptide fragment from STAT3.** The obtained $y^+$ and $b^+$ ion fragmentations are consistent with the hypothesis of covalent attachment through *O*-tosyl group displacement of S3I-201 onto Cys108.

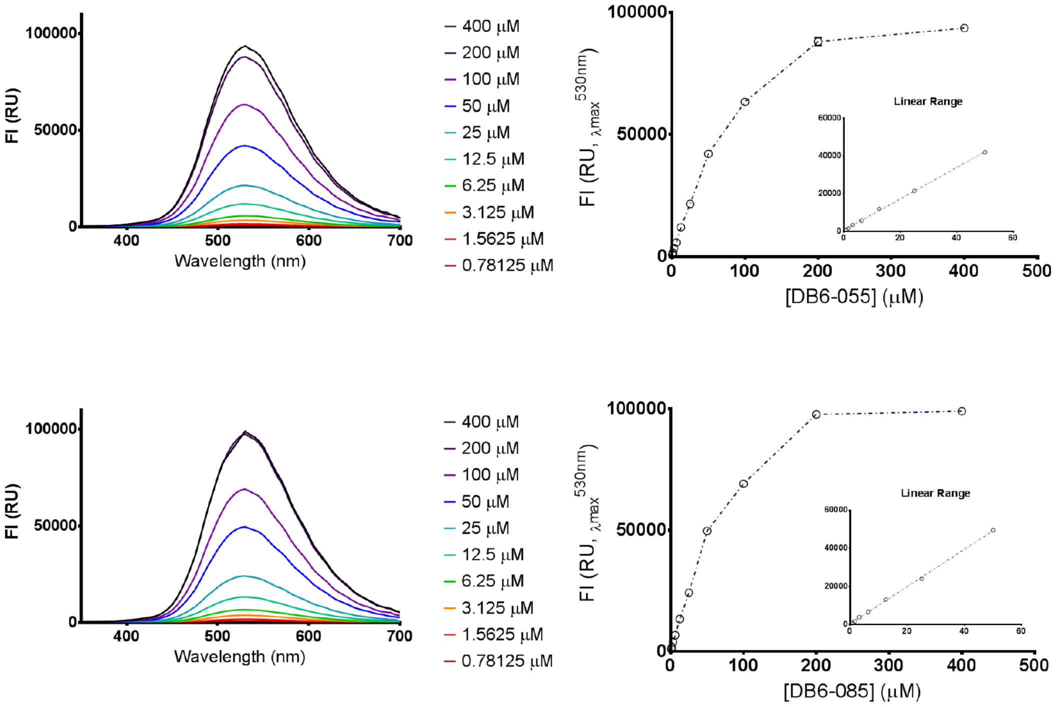


**Supplementary Figure S3: Fluorescence titration experiments of DB6-055 and DB6-085. A.** Gives the spectral scanning output from DB6-055 after excitation at 320 nm, tracking the concentration dependence of the fluorescent signal (left image, legend color coded for concentration). Relative fluorescence intensities of the 530 nm emission wavelengths from DB6-055 excitation were plotted and the linear portion of the spectral window highlighted (right image, insert). **B.** Gives the spectral scanning output from DB6-085 after excitation at 320 nm, tracking the concentration dependence of the fluorescent signal (left image, legend color coded for concentration). Relative fluorescence intensities of the 530 nm emission wavelengths from DB6-085 excitation were plotted and the linear portion of the spectral window highlighted (right image, insert).

**Supplementary Table S1: Linear regression analysis of the calibration curve obtained relating HPLC peak area to S3I-201 concentrations**

| | HPLC PA |
|---|---|
| Best-fit values | |
| Slope | 18.83 ± 0.1420 |
| Y-intercept when X=0.0 | 13.03 ± 12.31 |
| X-intercept when Y=0.0 | -0.6919 |
| 1/slope | 0.05310 |
| 95% Confidence Intervals | |
| Slope | 18.47 to 19.20 |
| Y-intercept when X=0.0 | -18.62 to 44.68 |
| X-intercept when Y=0.0 | -2.401 to 0.9774 |
| Goodness of Fit | |
| R square | 0.9997 |
| Sy.x | 25.96 |
| Is slope significantly non-zero? | |
| F | 17594 |
| DFn, DF | 1.000, 5.000 |
| P value | $< 0.0001$ |
| Deviation from zero? | Significant |
| Data | |
| Number of X values | 7 |
| Maximum number of Y replicates | 1 |
| Total number of values | 7 |
| Number of missing values | 0 |
| Equation | $Y = 18.83*X + 13.03$ |

**Supplementary Table S2: Non-linear regression analysis for the degradation of 100 µM SH-4-054, BP-1-102, SF-1-066, BP-1-102, and S3I-201 as measured in the presence of 10 mM GSH, 10 mM TCEP-HCl, 50 mM HEPES buffered to pH 7.4 and fit to a single exponential one phase decay model to obtain kinetic parameters $k_{obs}$ and $t_{1/2}$ values**

| | SH4-054 | BP1-102 | SF1-066 | BP5-087 | S3I-201 |
|---|---|---|---|---|---|
| One phase decay | | | Ambiguous | | |
| Best-fit values | | | | | |
| Y0 | 2169 | 1689 | 1518 | 2059 | 1508 |
| Plateau | 3.174 | 33.37 | 1540 | 1926 | 0.5750 |
| K | 0.5147 | 0.5322 | ~ 16.95 | 0.1348 | 2.735 |
| Half Life | 1.347 | 1.302 | ~ 0.04088 | 5.141 | 0.2535 |
| Tau | 1.943 | 1.879 | ~ 0.05898 | 7.417 | 0.3657 |
| Span | 2166 | 1656 | -21.92 | 133.4 | 1507 |
| Std. Error | | | | | |
| Y0 | 45.89 | 8.747 | 8.322 | 11.00 | 6.494 |
| Plateau | 17.12 | 3.861 | 3.146 | 18.97 | 3.508 |
| K | 0.02312 | 0.006806 | ~ | 0.04952 | 0.03079 |
| Span | 46.41 | 9.202 | 8.897 | 18.31 | 7.021 |
| 95% Confidence Intervals | | | | | |
| Y0 | 2077 to 2262 | 1671 to 1708 | 1501 to 1536 | 2036 to 2082 | 1495 to 1521 |
| Plateau | -31.22 to 37.57 | 25.29 to 41.45 | 1534 to 1547 | 1886 to 1965 | -6.665 to 7.815 |
| K | 0.4683 to 0.5612 | 0.5180 to 0.5465 | (Very wide) | 0.03183 to 0.2378 | 2.671 to 2.798 |
| Half Life | 1.235 to 1.480 | 1.268 to 1.338 | | 2.915 to 21.78 | 0.2477 to 0.2595 |
| Tau | 1.782 to 2.136 | 1.830 to 1.931 | | 4.205 to 31.42 | 0.3574 to 0.3744 |
| Span | 2073 to 2259 | 1637 to 1675 | -40.43 to -3.413 | 95.35 to 171.5 | 1493 to 1522 |
| Goodness of Fit | | | | | |
| Degrees of Freedom | 51 | 19 | 21 | 21 | 24 |
| R square | 0.9792 | 0.9994 | 0.2242 | 0.8117 | 0.9995 |
| Absolute Sum of Squares | 383949 | 3037 | 4364 | 8767 | 3150 |
| Sy.x | 86.77 | 12.64 | 14.41 | 20.43 | 11.46 |
| Constraints | | | | | |
| K | K > 0.0 | K > 0.0 | K > 0.0 | K > 0.0 | K > 0.0 |
| Number of points | | | | | |
| Analyzed | 54 | 22 | 24 | 24 | 27 |

**Supplementary Table S3: Non-linear regression analysis for the degradation of 100 µM S3I-201 and DB-5-112 as measured in the presence of 10 mM GSH, 10 mM TCEP-HCl, 50 mM HEPES buffered to pH 7.4 and fit to a single exponential one phase decay model to obtain kinetic parameters $k_{obs}$ and $t_{1/2}$ values**

| | S3I-201 | DB5-112 |
|---|---|---|
| One phase decay | | Interrupted |
| Best-fit values | | |
| Y0 | 1508 | 1892 |
| Plateau | 0.5750 | -435879 |
| K | 2.735 | 2.725e-005 |
| Half Life | 0.2535 | 25439 |
| Tau | 0.3657 | 36701 |
| Span | 1507 | 437770 |
| Std. Error | | |
| Y0 | 6.494 | |
| Plateau | 3.508 | |
| K | 0.03079 | |
| Span | 7.021 | |
| 95% Confidence Intervals | | |
| Y0 | 1495 to 1521 | |
| Plateau | -6.665 to 7.815 | |
| K | 2.671 to 2.798 | |
| Half Life | 0.2477 to 0.2595 | |
| Tau | 0.3574 to 0.3744 | |
| Span | 1493 to 1522 | |
| Goodness of Fit | | |
| Degrees of Freedom | 24 | |
| R square | 0.9995 | |
| Absolute Sum of Squares | 3150 | |
| Sy.x | 11.46 | |
| Constraints | | |
| K | K > 0.0 | K > 0.0 |
| Number of points | | |
| Analyzed | 27 | 16 |

**Supplementary Table S4: Non-linear regression analysis for the degradation of S3I-201 in the presence of variable concentrations of GSH in a 100 µM HEPES buffer at pH 7.4**

| | 0.00 mM | 0.50 mM | 1.00 mM | 2.00 mM | 5.00 mM | 10.00 mM |
|---|---|---|---|---|---|---|
| One phase decay | Ambiguous | | | | | |
| Best-fit values | | | | | | |
| Y0 | 99.73 | 100.2 | 86.93 | 99.37 | 108.3 | 98.76 |
| Plateau | ~ -1956 | 15.72 | 7.005 | 2.647 | 2.960 | 2.091 |
| K | ~ 0.0001114 | 0.09287 | 0.1660 | 0.2755 | 0.7566 | 1.583 |
| Half Life | ~ 6223 | 7.464 | 4.176 | 2.516 | 0.9162 | 0.4379 |
| Tau | ~ 8977 | 10.77 | 6.024 | 3.630 | 1.322 | 0.6317 |
| Span | ~ 2056 | 84.50 | 79.93 | 96.72 | 105.3 | 96.67 |
| Std. Error | | | | | | |
| Y0 | 0.2075 | 0.5493 | 0.5052 | 1.009 | 1.420 | 0.6245 |
| Plateau | ~ 530501 | 0.6512 | 0.5337 | 0.8340 | 1.065 | 0.3912 |
| K | ~ 0.02877 | 0.001974 | 0.003493 | 0.008324 | 0.02807 | 0.02679 |
| Span | ~ 530501 | 0.7063 | 0.5601 | 1.064 | 1.458 | 0.6633 |
| 95% Confidence Intervals | | | | | | |
| Y0 | 99.31 to 100.2 | 99.11 to 101.3 | 85.92 to 87.94 | 97.33 to 101.4 | 105.4 to 111.1 | 97.48 to 100.0 |
| Plateau | (Very wide) | 14.40 to 17.04 | 5.936 to 8.075 | 0.9663 to 4.329 | 0.8185 to 5.102 | 1.292 to 2.890 |
| K | (Very wide) | 0.08887 to 0.09686 | 0.1590 to 0.1730 | 0.2587 to 0.2923 | 0.7001 to 0.8130 | 1.528 to 1.638 |
| Half Life | (Very wide) | 7.156 to 7.799 | 4.007 to 4.360 | 2.372 to 2.679 | 0.8526 to 0.9900 | 0.4233 to 0.4536 |
| Tau | (Very wide) | 10.32 to 11.25 | 5.781 to 6.290 | 3.421 to 3.865 | 1.230 to 1.428 | 0.6106 to 0.6544 |
| Span | (Very wide) | 83.07 to 85.93 | 78.80 to 81.05 | 94.58 to 98.87 | 102.4 to 108.2 | 95.31 to 98.02 |
| Goodness of Fit | | | | | | |
| Degrees of Freedom | 30 | 39 | 57 | 45 | 50 | 30 |
| R square | 0.8797 | 0.9975 | 0.9974 | 0.9946 | 0.9905 | 0.9986 |
| Absolute Sum of Squares | 6.674 | 61.85 | 80.56 | 197.4 | 451.2 | 39.66 |
| Sy.x | 0.4717 | 1.259 | 1.189 | 2.094 | 3.004 | 1.150 |
| Constraints | | | | | | |
| K | K > 0.0 | K > 0.0 | K > 0.0 | K > 0.0 | K > 0.0 | K > 0.0 |
| Number of points | | | | | | |
| Analyzed | 33 | 42 | 60 | 48 | 53 | 33 |

The data were fit to a single exponential one phase decay model to obtain kinetic parameters $k_{obs}$ for the determination of the bimolecular rate constant and overall reaction order, with respect to GSH, for its reaction with S3I-201.

**Supplementary Table S5: Linear regression analysis correlating the observed rate constant ($k_{obs}$) to variable concentrations of the nucleophile GSH, under pseudo first order conditions, to determine the overall reaction order and true bimolecular rate constant ($k_2$) with respect to GSH for its reaction with S3I-201**

| | $k_{obs}$ ($h^{-1}$) |
|---|---|
| Best-fit values | |
| Slope | 0.1576 ± 0.003696 |
| Y-intercept when X=0.0 | -0.008510 ± 0.01886 |
| X-intercept when Y=0.0 | 0.05398 |
| 1/slope | 6.343 |
| 95% Confidence Intervals | |
| Slope | 0.1459 to 0.1694 |
| Y-intercept when X=0.0 | -0.06854 to 0.05152 |
| X-intercept when Y=0.0 | -0.3459 to 0.4130 |
| Goodness of Fit | |
| R square | 0.9984 |
| Sy.x | 0.02906 |
| Is slope significantly non-zero? | |
| F | 1819 |
| DFn, DFd | 1.000, 3.000 |
| P value | < 0.0001 |
| Deviation from zero? | Significant |
| Data | |
| Number of X values | 5 |
| Maximum number of Y replicates | 1 |
| Total number of values | 5 |
| Number of missing values | 1 |
| Equation | Y = 0.1576*X - 0.008510 |

**Supplementary Table S6: LC-MS/MS fragmentation matches (delta mass [ppm]) of alkylated peptide 108CLWEESR114 identified in *in vitro* alkylation reactions with S3I-201**

| #1 | $b^+$ | $b^{2+}$ | Seq. | $y^+$ | $y^{2+}$ | #2 |
|---|---|---|---|---|---|---|
| 1 | -483.9 | – | C-S3I-201 | | | 7 |
| 2 | +14.55 | – | L | -105.80 | +172.59 | 6 |
| 3 | -121.68 | – | W | -117.84 | -364.09 | 5 |
| 4 | -115.27 | -183.08 | E | -129.21 | – | 4 |
| 5 | -83.64 | – | E | -117.05 | – | 3 |
| 6 | -207.34 | -354.41 | S | -175.70 | – | 2 |
| 7 | | | R | -68.80 | – | 1 |

Observed and database-matched ions with the respective delta mass for the LC-MS/MS fragmentation patterns are highlighted in blue and red for $y^{n+}$ and $b^{n+}$ ions.

**Supplementary Table S7: LC-MS/MS fragmentation matches (delta mass [ppm]) of alkylated peptide 247QQIACIGGPPNICLDR262 identified in *in vitro* alkylation reactions with S3I-201**

| #1 | $b^+$ | $b^{2+}$ | Seq. | $y^+$ | $y^{2+}$ | #2 |
|---|---|---|---|---|---|---|
| 1 | – | – | Q | | | 16 |
| 2 | – | – | Q | – | – | 15 |
| 3 | -61.30 | – | I | -40.81 | -46.80 | 14 |
| 4 | -20.56 | – | A | -47.73 | -87.45 | 13 |
| 5 | -134.13 | – | -Carbamidometh | -62.21 | -119.41 | 12 |
| 6 | -11.53 | – | I | -69.07 | -223.47 | 11 |
| 7 | -148.06 | – | G | -87.42 | – | 10 |
| 8 | +46.02 | – | G | -46.45 | – | 9 |
| 9 | -113.04 | – | P | -79.30 | -337.89 | 8 |
| 10 | – | – | P | -141.96 | – | 7 |
| 11 | -96.99 | – | N | -140.86 | – | 6 |
| 12 | -35.49 | – | I | -74.79 | – | 5 |
| 13 | -101.42 | – | C-S3I-201 | -80.19 | – | 4 |
| 14 | -80.69 | -189.22 | L | -59.09 | – | 3 |
| 15 | -65.76 | – | D | -563.13 | – | 2 |
| 16 | | | R | – | – | 1 |

Observed and database-matched ions with the respective delta mass for the LC-MS/MS fragmentation patterns are highlighted in blue and red for $y^{n+}$ and $b^{n+}$ ions.

**Supplementary Table S8: LC-MS/MS fragmentation matches (delta mass [ppm]) of alkylated peptide 366VCIDKDSGDVAALR379 identified in *in vitro* alkylation reactions with S3I-201**

| #1 | $b^+$ | $b^{2+}$ | Seq. | $y^+$ | $y^{2+}$ | #2 |
|---|---|---|---|---|---|---|
| 1 | – | – | V | | | 14 |
| 2 | +251.53 | – | C-S3I-201 | – | – | 13 |
| 3 | -62.83 | – | I | -140.59 | -288.58 | 12 |
| 4 | +49.42 | – | D | -35.30 | +242.22 | 11 |
| 5 | – | – | K | -91.27 | -225.06 | 10 |
| 6 | -63.76 | +165.40 | D | -60.02 | +227.19 | 9 |
| 7 | +71.60 | – | S | -86.79 | – | 8 |
| 8 | – | -69.83 | G | -17.39 | +107.16 | 7 |
| 9 | -113.25 | – | D | -78.01 | – | 6 |
| 10 | -88.70 | – | V | -11.09 | +700.76 | 5 |
| 11 | -10.58 | +61.82 | A | +73.44 | – | 4 |
| 12 | – | – | A | +101.02 | – | 3 |
| 13 | – | – | L | -91.29 | – | 2 |
| 14 | | | R | – | – | 1 |

Observed and database-matched ions with the respective delta mass for the LC-MS/MS fragmentation patterns are highlighted in blue and red for $y^{n+}$ and $b^{n+}$ ions.

**Supplementary Table S9: LC-MS/MS fragmentation matches (delta mass [ppm]) of alkylated peptide 532LLGPGVNYSGCQITWAK548 identified in *in vitro* alkylation reactions with S3I-201**

| #1 | $b^+$ | $b^{2+}$ | Seq. | $y^+$ | $y^{2+}$ | #2 |
|---|---|---|---|---|---|---|
| 1 | – | – | L | | | 17 |
| 2 | – | – | L | – | – | 16 |
| 3 | – | – | G | -54.36 | – | 15 |
| 4 | – | – | P | -89.87 | – | 14 |
| 5 | -269.38 | – | G | – | – | 13 |
| 6 | -30.55 | – | V | – | – | 12 |
| 7 | -146.31 | – | N | -42.58 | – | 11 |
| 8 | +11.98 | – | Y | -125.20 | -174.04 | 10 |
| 9 | -155.14 | – | S | -136.83 | – | 9 |
| 10 | -100.58 | – | G | -63.55 | – | 8 |
| 11 | – | – | C-S3I-201 | -46.87 | – | 7 |
| 12 | – | – | Q | -232.11 | – | 6 |
| 13 | +21.09 | – | I | -244.92 | – | 5 |
| 14 | +56.49 | – | T | -143.96 | – | 4 |
| 15 | -89.30 | – | W | -136.95 | – | 3 |
| 16 | – | – | A | – | – | 2 |
| 17 | | | K | – | – | 1 |

Observed and database-matched ions with the respective delta mass for the LC-MS/MS fragmentation patterns are highlighted in blue and red for $y^{n+}$ and $b^{n+}$ ions.

**Supplementary Table S10: LC-MS/MS fragmentation matches (delta mass [ppm]) of alkylated peptide 686YCRPESQEHPEADPGSAAPYLK707 identified in *in vitro* alkylation reactions with S3I-201**

| #1 | $b^{+1}$ | $b^{2+}$ | $b^{3+}$ | Seq. | $y^{+}$ | $y^{2+}$ | $y^{3+}$ | #2 |
|---|---|---|---|---|---|---|---|---|
| 1 | – | – | – | Y | | | | 22 |
| 2 | -5.46 | – | – | C-S3I-201 | – | – | -72.29 | 21 |
| 3 | -281.44 | – | – | R | – | – | +166.84 | 20 |
| 4 | – | – | – | P | – | – | – | 19 |
| 5 | -232.37 | – | – | E | – | – | – | 18 |
| 6 | -190.98 | -286.04 | – | S | – | – | – | 17 |
| 7 | – | – | – | Q | – | – | – | 16 |
| 8 | -136.91 | – | – | E | – | -195.36 | – | 15 |
| 9 | – | – | – | H | – | -52.07 | – | 14 |
| 10 | – | +60.78 | – | P | -144.12 | – | – | 13 |
| 11 | – | – | +152.03 | E | – | – | – | 12 |
| 12 | – | – | – | A | -125.99 | – | – | 11 |
| 13 | – | – | – | D | – | – | – | 10 |
| 14 | – | – | +269.03 | P | -149.01 | – | – | 9 |
| 15 | – | – | -254.86 | G | +26.46 | – | – | 8 |
| 16 | – | – | – | S | -159.80 | – | – | 7 |
| 17 | – | – | -165.32 | A | -149.73 | – | – | 6 |
| 18 | – | – | – | A | -68.23 | – | – | 5 |
| 19 | – | – | -237.17 | P | -165.32 | – | – | 4 |
| 20 | – | -5.93 | – | Y | – | – | – | 3 |
| 21 | – | – | +120.11 | L | -55.30 | – | – | 2 |
| 22 | | | | K | – | – | – | 1 |

Observed and database-matched ions with the respective delta mass for the LC-MS/MS fragmentation patterns are highlighted in blue and red for $y^{n+}$ and $b^{n+}$ ions.
